# miRDB: an online resource for microRNA target prediction and functional annotations

**DOI:** 10.1093/nar/gku1104

**Published:** 2014-11-05

**Authors:** Nathan Wong, Xiaowei Wang

**Affiliations:** 1 Department of Biomedical Engineering, Washington University, St. Louis, MO 63130, USA; 2 Department of Radiation Oncology, Washington University School of Medicine, St. Louis, MO 63108, USA

## Abstract

MicroRNAs (miRNAs) are small non-coding RNAs that are extensively involved in many physiological and disease processes. One major challenge in miRNA studies is the identification of genes regulated by miRNAs. To this end, we have developed an online resource, miRDB (http://mirdb.org), for miRNA target prediction and functional annotations. Here, we describe recently updated features of miRDB, including 2.1 million predicted gene targets regulated by 6709 miRNAs. In addition to presenting precompiled prediction data, a new feature is the web server interface that allows submission of user-provided sequences for miRNA target prediction. In this way, users have the flexibility to study any custom miRNAs or target genes of interest. Another major update of miRDB is related to functional miRNA annotations. Although thousands of miRNAs have been identified, many of the reported miRNAs are not likely to play active functional roles or may even have been falsely identified as miRNAs from high-throughput studies. To address this issue, we have performed combined computational analyses and literature mining, and identified 568 and 452 functional miRNAs in humans and mice, respectively. These miRNAs, as well as associated functional annotations, are presented in the FuncMir Collection in miRDB.

## INTRODUCTION

MicroRNAs (miRNAs) are a family of small non-coding RNAs that play important regulatory roles in many physiological and disease processes (1,2). About 2000 human miRNAs have been discovered to date, and collectively these miRNAs regulate the expression of thousands of protein-coding gene targets at both post-transcriptional and translational levels (3–5). miRNAs exert their functions via target downregulation. Thus, identification of gene targets is critical for functional characterization of miRNAs. Currently, experimental identification of miRNA targets is a time-consuming process and as a result most researchers rely on computational tools to initially identify a set of candidate gene targets for further experimental characterization. To facilitate the process of selecting functional targets at the genome level, we have previously developed an online resource, miRDB for miRNA target prediction and functional annotations (6). Here, we present major updates to miRDB, including updated target prediction data, a new web server interface for custom target prediction, as well as the inclusion of a set of functional miRNAs annotated by integrating computational analyses with literature mining. The web interface of miRDB has also been updated to accommodate these new database features at http://mirdb.org/.

## METHODS AND RESULTS

### Updates on miRNA target prediction data

Since the first version of miRDB was established in 2008, thousands of novel miRNAs have been discovered. In addition, annotations of gene targets, especially concerning the identification of 3′-UTR sequences, have been significantly expanded. Thus, we have performed a major update on target prediction data by employing the up-to-date miRNA and target gene annotations. All miRNA sequences and annotations were downloaded from miRBase (version 21) in June 2014 (7). We have adopted the NCBI RefSeq database for identification of 3′-UTR sequences. In brief, RefSeq sequences were downloaded from NCBI's ftp site (8) and further parsed with the BioPerl program to obtain the 3′-UTR sequences of the transcripts. Target prediction was then performed with the MirTarget algorithm, which was developed by analyzing high-throughput expression profiling data in a support vector machine framework (9). Unlike most other prediction algorithms, MirTarget predicts both conserved and nonconserved gene targets by treating target site conservation as an important but non-required sequence feature. The robust performance of MirTarget has been extensively demonstrated. For example, a recent independent analysis shows that MirTarget has superior performance over other public algorithms for identifying miRNA-downregulated gene targets (10). In this miRDB update, we have also updated the MirTarget algorithm by including additional model training data, which were generated from miRNA-target pairs experimentally identified by RNA-seq (11). Details of the algorithmic improvement will be described elsewhere.

**Figure 1.**
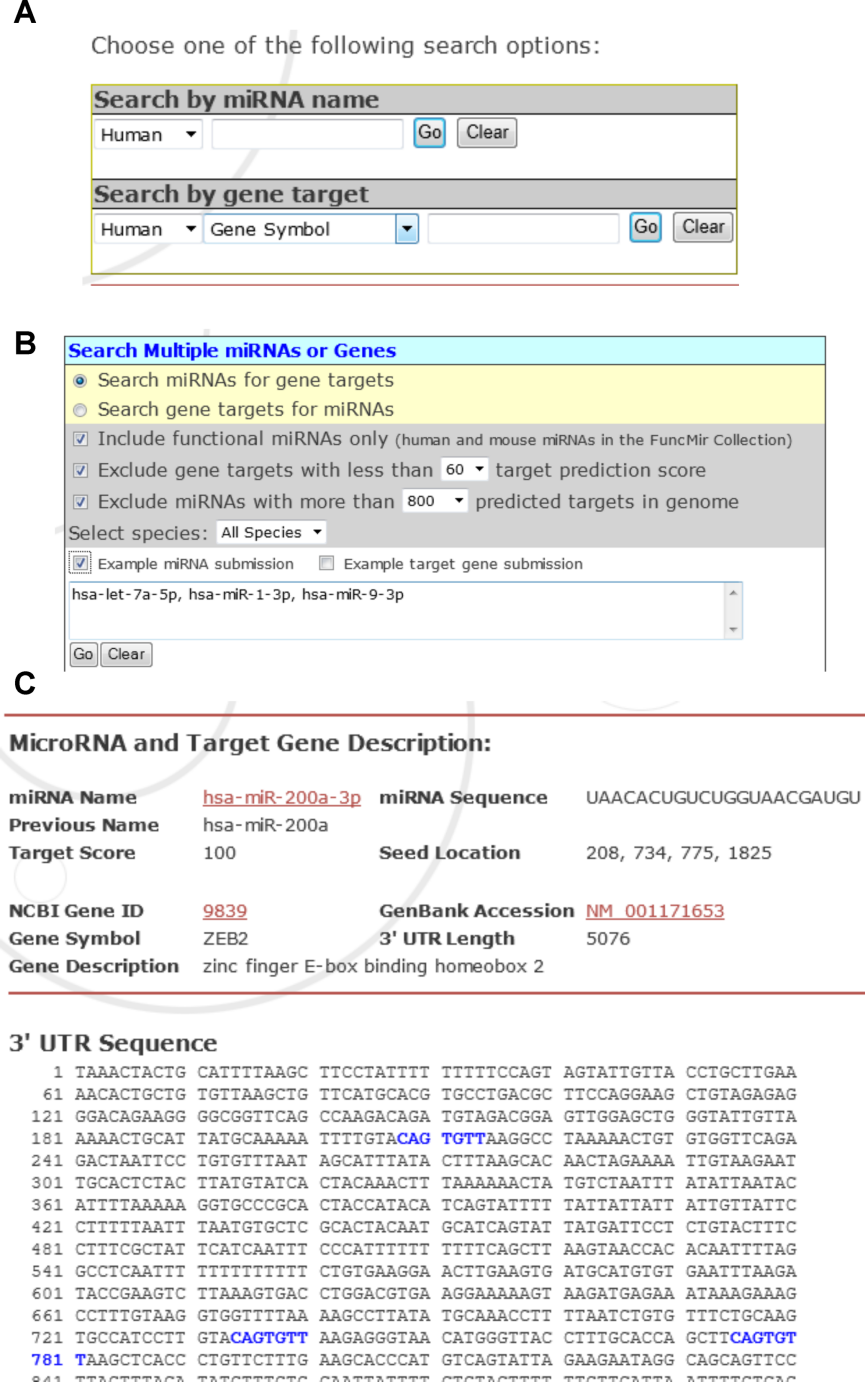
miRDB web interface for miRNA target prediction. (**A**) Search for miRNA targets by a single miRNA or gene target. (**B**) Search for miRNA targets by multiple miRNAs or gene targets. (**C**) A screenshot of target prediction data retrieved from miRDB.

With updated genomic data and the MirTarget algorithm, we have performed genome-wide miRNA target prediction for all known transcripts (including all isoforms) from five species—human, mouse, rat, dog and chicken. In total, 2.1 million gene targets were predicted to be regulated by 6709 miRNAs in these five species. All the targets have a prediction score in the range of 50–100 as assigned by MirTarget, with a higher score representing more statistical confidence in the prediction result. Detailed statistics of the target prediction are presented in the miRDB website. All the target prediction data as well as the associated genomic annotations were imported into a backend MySQL database for web presentation. The users can search for precompiled results via miRDB web interface, using either miRNA or gene target search terms. Notably, the users have the flexibility of searching a single miRNA/gene target (Figure 1A), or a combination of multiple miRNAs/gene targets (Figure 1B). The users can download target prediction results for individual miRNAs or gene targets to a tab-delimited spreadsheet file via the Target Mining search interface. In addition, the users can download all precompiled target prediction data via the miRDB download page. A representative target prediction result retrieved from miRDB is presented in Figure 1C. There has been a major change in miRNA naming rules recently, resulting in multiple names describing the same miRNA (7). Thus, historical names for the same miRNAs are also presented in the result page. Data stored in miRDB are interconnected with the miRBase database (7). In each miRBase miRNA entry, there is a dynamic link directing to specific miRNA target prediction data in miRDB. The miRDB web interface and backend database are hosted by a Linux server at Washington University (http://mirdb.wustl.edu). Besides searching for target sites in 3′-UTR, the users may also locate unconventional target sites in the coding region or 5′-UTR via the Custom Prediction web interface.

### Web server interface for custom miRNA target prediction

**Figure 2.**
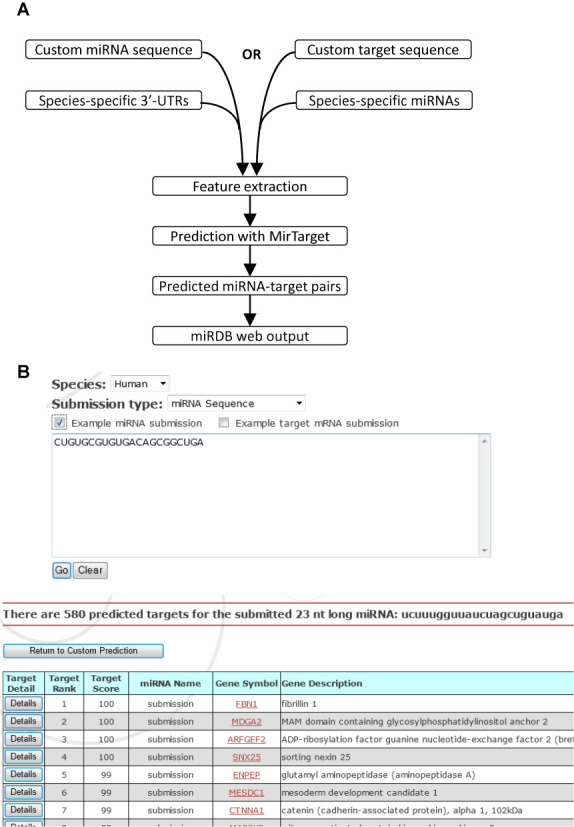
Custom target prediction with user-provided sequences. (**A**) Workflow of the target prediction pipeline for the miRDB web server. (**B**) A screenshot of the custom web search interface and a representative result page.

One major update of miRDB is the implementation of the web server function for custom target prediction. With this new function, users are now able to provide their own sequences (either a miRNA or target sequence) for target prediction. Specifically, the user can choose between providing a miRNA sequence (17–30 nt) or a target gene sequence (100–30 000 nt) for prediction of miRNA targets in one of the five species—human, mouse, rat, dog or chicken. On submission, the server calls a backend Perl script which implements the MirTarget prediction algorithm (Figure 2A). The script takes as its inputs the user-provided sequence, as well as the selected species in which the prediction is to be conducted. For miRNA sequence submissions, a target sequence file, containing the 3′-UTR sequences from all known genes in a given species, is loaded into the computational pipeline. These 3′-UTR sequences were obtained by parsing full-length NCBI RefSeq transcript sequences. In the event of a target sequence submission, the server imports all the species-specific miRNAs, as collated from miRBase v21.

For either custom miRNAs or custom targets, the program first evaluates each potential miRNA-target pair by initially screening for 7-mer seed complementarity in the target site. Based on the relative strength of each sequence-based target recognition feature, the MirTarget algorithm is then used to predict miRNA targets. MirTarget was originally developed for genome-wide target prediction, and it has been customized here to become an integral component of the web server pipeline. When the prediction process is completed, the user progresses to the results page. The prediction data are relayed to a Perl-CGI script for web presentation. In addition, the script also retrieves relevant gene annotations for each target from a backend MySQL database. The web page then displays the results in a manner similar to those produced by a standard miRDB query. Briefly, the scores are sorted in descending order and show the user the ranking of the miRNA-target pair, the score, the names of the miRNA and the target, and in the case of custom miRNA submission, the annotations of the gene targets. Additionally, users have the option of viewing more details of the target prediction, which includes both the miRNA and the target sequences and the locations of seed matches (Figure 2B). Concerning the speed of the prediction process, it takes about 5–10 s per query for genome-wide target prediction for a user-provided miRNA sequence; it takes about 1–2 s per query for a typical user-provided target sequence (∼2 kb long).

### FuncMir collection for annotating functional miRNAs

The discovery of a large number of miRNAs presents grand new opportunities to uncover the complexity of gene expression regulatory networks (12). On the other hand, many of the newly discovered miRNAs are not likely to be functionally relevant, or may even have been false discoveries resulting from low-quality data. For example, an RNA-seq experiment, if not properly performed or analyzed, could potentially produce hundreds of falsely discovered ‘novel’ miRNAs. Unfortunately, many such ‘novel’ miRNAs might have already been included in miRBase, which only implements limited quality control filters and in principle allows the submission of any miRNAs that have been reported in peer-reviewed studies (7). As a result, a large number of miRNAs included in miRBase are not likely to play any functional roles in gene expression regulation. Our inability to distinguish functional miRNAs from non-functional ones could severely compromise both computational and experimental studies on miRNA functions, leading to unnecessary wastes of resources or even obscure real important findings. For example, a functional library screen will be negatively impacted by the inclusion of many falsely discovered non-functional miRNAs. On the computational side, the inclusion of many non-functional miRNAs can severely reduce the significance of real biological findings, such as those on functional miRNAs that regulate competing endogenous RNAs (ceRNAs) (13).

Aware of these pitfalls, multiple groups have developed new strategies to identify authentic functional miRNAs (7,14–17). For example, a recent miRBase publication has presented stringent computational criteria to select 278 and 370 ‘High Confidence’ miRNAs for humans and mice, respectively, based on structural features of precursor miRNA processing and RNA-seq expression data (7). However, current knowledge on miRNA biogenesis is still very limited and there are many well-characterized authentic miRNAs that failed the computational selection process.

In this miRDB update, we present a new strategy to identify functional miRNAs by combining computational analysis with literature mining. The four selection criteria for functional miRNAs are described below.

- *PubMed literature mining.* We took advantage of the self-correcting nature of scientific research, as false discoveries are generally corrected or ignored by the research community while true discoveries are repeatedly validated and further explored by independent studies. This concept was adopted to identify miRNAs that are of functional importance. To this end, the NCBI PubMed database (http://www.ncbi.nlm.nih.gov/pubmed) was mined to retrieve articles that are relevant to human and mouse miRNAs. Mapping index data from the NCBI Gene database (http://www.ncbi.nlm.nih.gov/gene) were used to associate specific miRNAs to corresponding PubMed records. Among all 1872 human miRNAs, 716 were described in at least two independent studies, 454 in at least three independent studies and 338 in at least four independent studies.
- *Sequence conservation.* Conserved miRNAs across multiple species are likely to be functionally important. Thus, miRNA sequences across five vertebrate species were compared, including human, mouse, rat, dog and chicken. All the miRNA sequences were retrieved from miRBase. A mature miRNA is considered to be conserved if its ortholog is found in at least one other species. Since the 3′-end of a mature miRNA is often post-transcriptionally modified and becomes more variable, perfect match of only the 5′-end 19 nt of a mature miRNA was required to define orthologous miRNAs. In this way, 323 conserved precursor miRNAs (encoding conserved mature miRNAs) in humans were identified.
- *Expression profile.* Public RNA-seq data were used to profile miRNA expression levels. Read counts from 81 RNA-seq experiments have previously been summarized and associated with the miRNAs by miRBase. This miRBase expression dataset was downloaded and further normalized for functional miRNA identification.
- *Functional annotations by miRBase.* In a recent miRBase update, 278 ‘High Confidence’ human miRNAs were identified based on structural analysis of precursor miRNAs combined with expression counts (7). The High Confidence status of a miRNA was used as one criterion for functional miRNA identification.

### Composite scores to identify functional miRNAs

Four individual criterion scores were calculated to identify functional miRNAs. (i) *PubMed Score* based on the number of associated PubMed records for each miRNA, with score of 1 for two PubMed records, 2 for three records and 3 for four or more records. (ii) *Conservation Score* based on the level of conservation, with score of 1 for one orthologous miRNA in another species and 2 for two or more orthologous miRNAs. (iii) *Expression Score* based on normalized read counts from 81 RNA-seq experiments, with score of 1 for 1–10 reads per million reads, and 2 for over 10 reads per million reads. (iv) *miRBase Score*, with score of 1 if a miRNA has previously been classified as a High Confidence miRNA by miRBase.

**Figure 3.**
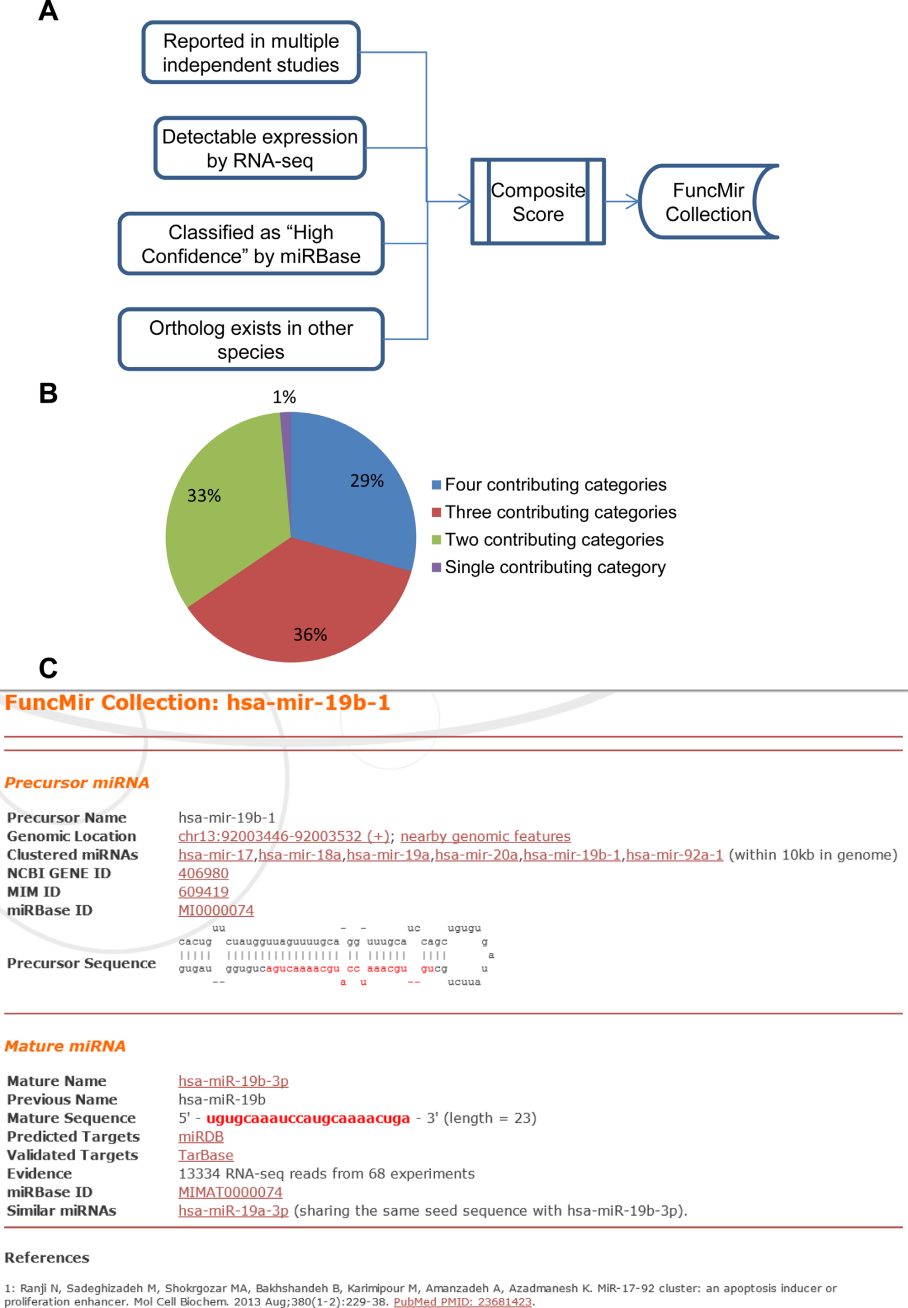
Identification of functional miRNAs by combinatorial analysis of four selection criteria. (**A**) Composite scores were calculated by summarizing individual criterion scores from four categories. All functional miRNAs have a composite score ≥3. (**B**) Distribution of functional human miRNAs identified by various numbers of scoring criteria. (**C**) A screenshot of a FuncMir miRNA homepage. For each functional miRNA, a web page is established by integrating data from various sources, including miRBase, PubMed and NCBI Gene databases. The page for hsa-mir-19b-1 is presented here as an example.

A composite score was calculated for each miRNA by summarizing four individual criterion scores described above (Figure 3A). A score of 3 or higher was used to define functional miRNAs. In this way, 568 and 452 functional precursor miRNAs were identified for humans and mice, respectively, which represent about one-third of all miRBase entries for the two species. Interestingly, the four individual criteria identified largely overlapping miRNAs. Only 1% of all functional miRNAs were identified by a single criterion only (PubMed Score), whereas two-thirds were identified by at least three of the four criteria (Figure 3B). The relative contribution of each criterion was also evaluated by removing the corresponding individual criterion score from composite score calculation. PubMed Score had the largest contribution, and was required for the identification of 183 (32%) functional miRNAs in humans. Similarly, Expression Score, miRBase Score and Conservation Score were required for the identification of 165, 29 and 20 functional human miRNAs, respectively. The complete list of both composite scores and individual criterion scores for all the miRNAs is presented in Supplementary File 1. For each functional precursor miRNA, the dominantly expressed mature miRNA was selected. In some cases, a second mature miRNA was selected from the same precursor if its expression level was no less than 10% of that of the dominant mature miRNA and its read count was no less than 5 reads per million reads as determined by RNA-seq experiments. In this way, 654 and 442 functional mature miRNAs were identified in humans and mice, respectively. All these identified functional miRNAs were included in the FuncMir Collection of miRDB, with one entry homepage for each miRNA. A screenshot of a representative miRNA entry is presented in Figure 3C. The CLASH RNA-seq data (11) were analyzed to evaluate the expression profiles of FuncMir miRNAs. Among all 2588 human mature miRNAs included in miRBase, 322 were detected at least five times per million sequence reads in HEK293 cells. Most of these expressed miRNAs (79.2%) have been included in the FuncMir Collection. In summary, the FuncMir Collection helps to facilitate researchers to focus on functionally relevant miRNAs, especially in experimental screens for miRNA functions or systematic computational analysis at the genome level.

## SUPPLEMENTARY DATA

Supplementary Data are available at NAR Online.
